# 3D Cell Printing and Manipulation with Magnetic Bioinks

**DOI:** 10.3390/biomedicines14061311

**Published:** 2026-06-09

**Authors:** Sarah Mishriki, Tamaghna Gupta, Rakesh P. Sahu, Ishwar K. Puri

**Affiliations:** 1 School of Biomedical Engineering, McMaster University, Hamilton, ON L8S 4L8, Canada; 2 Department of Orthopaedic Surgery, Stanford University, Stanford, CA 94063, USA; 3 Department of Materials Science and Engineering, McMaster University, Hamilton, ON L8S 4L8, Canada; 4 Department of Mechanical Engineering, McMaster University, Hamilton, ON L8S 4L8, Canada; 5 Department of Aerospace and Mechanical Engineering, University of Southern California, Los Angeles, CA 90089, USA; 6 Mork Family Department of Chemical Engineering and Materials Science, University of Southern California, Los Angeles, CA 90089, USA; 7 Alfred E. Mann Department of Biomedical Engineering, University of Southern California, Los Angeles, CA 90089, USA

**Keywords:** magnetic bioink, magnetic 3D bioprinting, magnetic susceptibility, label-based manipulation, label-free manipulation, positive magnetophoresis, negative magnetophoresis

## Abstract

Three-dimensional (3D) cell culture models more faithfully reproduce native tissue organization and function than conventional two-dimensional systems, yet many existing bioprinting methods depend on scaffolds, complex instrumentation, or limited control over cell positioning. This review examines magnetic bioinks as a versatile platform for contactless 3D cell manipulation and biofabrication. It first outlines the fundamentals of magnetophoresis and defines magnetic bioinks as combinations of magnetic agents, including magnetic nanoparticles or paramagnetic salts, with biological components such as cells, proteins, or fluids. The review then compares label-based strategies, in which cells are magnetized and guided by positive magnetophoresis, with label-free approaches that exploit magnetic susceptibility differences to position diamagnetic cells through negative magnetophoresis. Across these methods, magnetic bioinks have enabled single-cell sorting, spatial patterning, spheroid and co-culture assembly, multilayer tissue formation, and hydrogel-integrated printing. These capabilities support applications in disease modeling, drug screening, biosensing, regenerative medicine, and emerging biofabrication under microgravity conditions. The paper also highlights key limitations, including nanoparticle biocompatibility, paramagnetic salt toxicity, osmotic stress, and the need for better assay standardization and translational validation. Overall, magnetic bioinks represent a promising scaffold-free approach for rapidly producing physiologically relevant 3D biological constructs for research and clinical innovation.

## 1. Introduction

Human diseases are often first studied in a population of mammalian cells adhered to an artificial two-dimensional (2D) substrate. Even when human cells are used, an in vitro environment can lead to inaccurate conclusions. After 2D cell culture investigations, animal studies are used to evaluate therapeutic candidates in vivo [1], but 2D cell models are not appropriate indicators of preclinical success. Animal models can have different cell receptors [2] and non-human pathophysiology [3]. Hence, approximately only 8% of therapeutic oncology candidates studied in animal models have been translated into clinical human trials. Ethical concerns, standardization issues, and high costs also limit the use of animal models. While mathematical models can predict clinical outcomes of tumorigenesis [4] and chemotherapeutic drug efficacy [5], they do not consider the influence of a dynamic biological system with feedback.

Due to shortcomings associated with 2D cell models, animal studies, and analytical solutions, three-dimensional (3D) cell models can be more appropriate as disease models. 3D cultures demonstrate superior in vivo phenomena, such as gene expression [6], drug potency [1], cell morphology [7,8], formation of organized extracellular matrix (ECM) [8], and regions of proliferative, senescent, and necrotic cell layers [9]. These cultures better reproduce the mechanical properties mediated by neighboring cells and the ECM [7]. Since the goal of 3D cell models is to represent native tissue environments in vitro, it follows that the development of 3D cellular structures will eventually replace 2D cell models to refine and reduce the number of animal studies for high-throughput screening [10]. Other applications of 3D cellular structures include drug discovery and screening [8,11,12,13,14], disease modeling with organoids [15,16,17], organ-on-chip [18,19], tumor-on-chip [20,21] tumor modeling [22], and transplantable tissues [23].

The static definition of 3D printing can be broadened to include emerging printing techniques that also exhibit dynamic, time-dependent changes in the biological constructs. When a stimulus-responsive material is used [24,25], the construct can be controlled over time [26]. Various printing methods have been employed to manipulate cells in a 3D space using acoustics [27,28], temperature [29,30], light [31], biochemical gradients [32], and electrical stimuli [33], where magnetic field-based techniques are recent additions [34,35,36,37,38,39,40]. Using action-from-a-distance with a magnetic field, high-resolution patterns can be printed with magnetic ink, often without introducing scaffolds or solid structures. Magnetic bioinks are suspensions containing magnetic materials that control the migration of biological entities under the influence of an applied magnetic field, a phenomenon known as magnetophoresis. This definition is consistent with traditional concepts of 3D printing, which offers controlled positioning of suspended entities. Simply put, magnetic manipulation and printing require magnetic fields and magnetizable materials that respond to the fields. These fields do not require complex instrumentation and can be generated with common rare-earth magnets [41]. Thus, magnetic manipulation is easily integrated with existing systems for contactless control of target specimens.

This review focuses on the recent advances in magnetic bioinks composed of magnetic salts, magnetic nanoparticles, and cells for applications in cell manipulation and 3D cell printing. A summary of manipulation strategies using magnetic bioinks is provided in Table 1. The ability to form 3D cellular constructs with physiological relevance is based on the rich science of recent innovations in manipulation techniques using magnetic materials. Special attention is placed on the emerging use of magnetic bioinks to print 3D cellular structures and tissues that offer solutions for diagnosis, in vitro research, and treatment of diseases.

## 2. Magnetophoresis Fundamentals

Magnetic fields arise due to the motion of electric charges in the form of free and bound currents. The magnetic field, **B**, within a magnetizable material is expressed as

**Figure 1:**
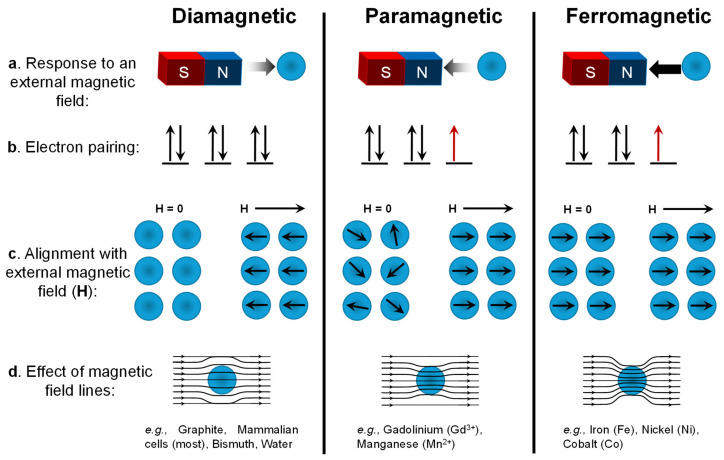
**Characterization of diamagnetic, paramagnetic and ferromagnetic materials.** (**a**) Response to an external, inhomogeneous magnetic field: weakly repelled by, weakly attracted to, and strongly attracted to respectively; (**b**) electron pairing: paired, contain at least one unpaired, and contain at least one unpaired, respectively; (**c**) alignment with an externally applied magnetic field: magnetization is in a direction opposite to that of **H**, magnetization is aligned with **H**, and magnetization is aligned with **H**, respectively; and (**d**) magnetic field lines: are repelled, are weakly attracted, are strongly attracted, respectively.

**B** = *μ*_0_ (**M** + **H)**, (1)

where *μ*_0_ = (4π × 10^−7^ N/A^2^) denotes the permeability of vacuum. A free current, i.e., a flow of free charges through a conductor, produces **H**. The magnetization **M**, a material property, is produced by the collection of bound circulating currents generated by the orbital motion of electrons and electron spin [60]. The bound current loops behave as magnetic dipoles, and, in most materials, their orientation is random in the absence of an external magnetic field. When a magnetic field is imposed, materials can acquire a net magnetization parallel (paramagnets) or opposite (diamagnets) to the imposed field. In weak magnetic fields (**B** ≤ 6 mT), the magnetization of most materials is proportional to the imposed **H** [61], i.e.,

**M** = *χ_i_***H**, (2)

where *χ_i_* denotes the intrinsic magnetic susceptibility, a dimensionless quantity that is positive for paramagnetic and negative for diamagnetic materials. Materials can be characterized by their response to a magnetic field as diamagnetic, paramagnetic, or ferromagnetic, as shown in Figure 1.

**Diamagnetic materials** are weakly repelled by a magnetic field and have a negative magnetic susceptibility, χ (~10^−6^–10^−3^) [62]. They do not contain any unpaired electrons and have a net magnetic moment of zero in the absence of an external field **H**. In the presence of an external magnetic field, their magnetization is in a direction opposite to that of **H**, therefore repelling the magnetic lines of force [63]. An example of a diamagnetic material is graphite [64], which is a lattice structure produced by sp^2^-hybridized carbon atoms. Other diamagnetic materials include bismuth [62], brass [62], and most cells [65,66,67].

**Paramagnetic materials** are weakly attracted towards a magnetic field and have a small positive magnetic susceptibility (χ ~ 10^−6^–10^−1^) [62]. They contain at least one unpaired electron at the atomic level and have randomly aligned magnetic moments in the absence of a magnetic field. In the presence of a magnetic field, their magnetic moments align in the direction of **H** and are weakly attracted along the magnetic lines of force [63]. Like diamagnetic materials, their magnetic behavior is also diminished in the absence of a magnetic field. Examples include transition metal complexes such as chelates of gadolinium ions (Gd^3+^), manganese (Mn^3+^), and magnesium (Mg^2+^) that are used to magnetize biological inks for label-free manipulation.

**Ferromagnetic materials** retain their own magnetic field even after the external magnetic field has been removed. Like paramagnetic materials, ferromagnetic materials have unpaired electrons and attract magnetic field lines. In the absence of a magnetic field, their magnetic moments are aligned and parallel to one another, which enhances their magnetization and attracts them strongly along the magnetic lines of force. When an external magnetic field is applied, the magnetic moments align with the direction of **H** [62]. These materials and their alloys are often used as permanent magnets, e.g., neodymium (Nd) [56,58,59,68,69,70], nickel (Ni) [71], and cobalt (Co) [72]. When the size of the ferromagnetic materials is in the range of nanometers (nm), the magnetization of magnetic domain of each particle flips randomly resulting in a zero-residual magnetization. However, in the presence of an external magnetic field the magnetic domains align in the direction of the applied field. This state of ferromagnetic materials is called superparamagnetism [73,74].

When a magnetizable material is exposed to a non-uniform magnetic field, it experiences a magnetic body force, also known as Kelvin body force, resulting from the interaction between the imposed **H** and induced **M**. The total magnetic body force acting on a finite-sized magnetizable body [75],

(3) $$F=∭\left[\mu_{0}(M\cdot \nabla )H+\nabla (\frac{1}{2}\mu_{0}M\cdot M\right)]d\vartheta ,$$

where dϑ is the differential volume element. Using the corollary of the Gauss divergence theorem, $∭\nabla (\frac{1}{2}\mu_{0}M\cdot M)d\vartheta =\frac{1}{2}\mu_{0}\;∯M\cdot M\;ds$, where d**s** is the differential area vector on the control surface. For a magnetizable body surrounded by a nonmagnetic medium, the control surface that completely encloses the magnetizable body lies in a region outside the body where **M** = 0. Thus, the term $\frac{1}{2}\mu_{0}\;∯M\cdot M\;ds$ vanishes and Equation (3) reduces to:

(4) $$F=\mu_{0}∭\left(M\cdot \nabla \right)Hd\vartheta .$$

Physically, this force expression implies that the magnetizable body is attracted towards regions of higher **H**. Substituting Equation (2) in Equation (4), the magnetic force on an isolated spherical magnetic particle of radius *a* is obtained as:

(5) $$F=\mu_{0}(\frac{4}{3}\pi a^{3})\chi_{eff}(H_{0}\cdot \nabla )H_{0}.$$

Here, $\chi_{eff}$ is the effective magnetic susceptibility of the particle, which is related to the intrinsic susceptibility $\chi_{i}$ by [76]

(6) $$\chi_{eff}=\frac{\chi_{i}}{1+N\chi_{i}},$$

where *N* is the demagnetization factor. For a sphere, *N* = $\frac{1}{3}$. The effective magnetic susceptibility $\chi_{eff}$ considers the demagnetization effect when the induced particle dipole moment distorts the imposed field. The field $H_{0}$ is located at the particle center. It is assumed that the particle is small enough so that the magnetic field and its gradient do not vary significantly within the particle volume.

Equation (5) neglects the magnetic susceptibility of the surrounding medium. This equation is used to evaluate the Kelvin body force acting on ferromagnetic or superparamagnetic particles suspended in a nonmagnetic fluid. The surrounding medium’s magnetic susceptibility is at least four orders of magnitude smaller than the magnetic particles and is neglected [61]. For cases where the susceptibility of the medium and the suspended particles are comparable (e.g., diamagnetic particles suspended in a paramagnetic medium), Equation (5) assumes the form:

(7) $$F=\mu_{0}(\frac{4}{3}\pi a^{3})∆\chi (H_{0}\cdot \nabla )H_{0},$$

where ∆χ = $\chi_{p}-\chi_{m}$ denotes the difference between the magnetic susceptibilities of the particle ($\chi_{p}$) and the surrounding medium ($\chi_{m}$). For diamagnetic particles ($\chi_{p}<0$) suspended in a paramagnetic medium ($\chi_{m}>0$), ∆χ is negative. Hence, the Kelvin body force reverses so that diamagnetic particles are transported to the magnetic field minima, also known as negative magnetophoresis.

## 3. 3D Cellular Assembly Techniques

The development of in vitro 3D cellular models is often inspired by an unmet medical need. For example, the consequences of burns and tissue removal inspired the formation of adipogenic-differentiated 3D cellular structures for clinical applications and as models for obesity-related pathology [77]. Prefabricated 3D cellular structures composed of monotypic or co-culture assemblies can be used as building blocks for larger tissue or organ printing [41,78,79].

Common methods to engineer tissues in vitro typically require 3D bioprinting, which prints cell-laden hydrogels and permanent or sacrificial biological scaffolds for subsequent cell seeding. An ideal scaffold must be non-toxic to the cells, non-immunogenic (in case it is implanted), have satisfactory mechanical properties, and facilitate appropriate tissue growth and differentiation [80]. However, the scaffold-based approach is expensive and requires trained personnel for operation and rigorous troubleshooting to optimize bioink printability. Scaffold-based techniques can produce poor cell viability, compromise the mechanical properties of the tissue construct, and lead to poor cell compatibility during gelation [81].

Alternatively, gravity-based approaches such as the hanging drop and rounded non-adherent surface-based methods produce 3D cell clusters without a scaffold and rely on intercellular interactions to produce ECM. However, these techniques provide limited control over the spatial organization of cells in the assembled constructs.

**Figure 2:**
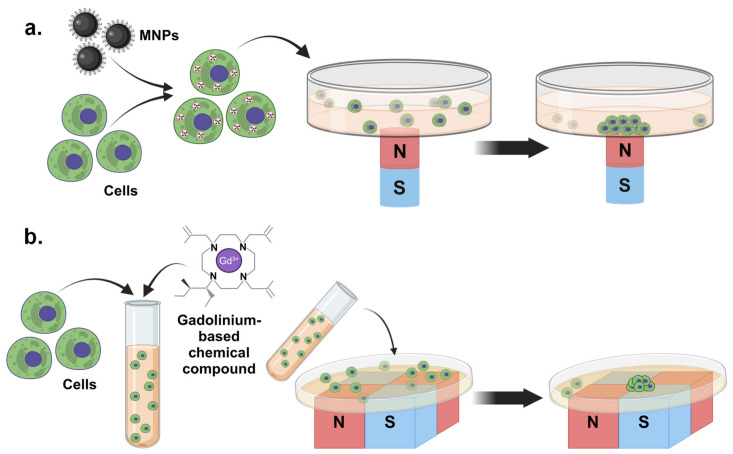
Printing of 3D cellular structures using magnetic cell bioinks. (**a**) Magnetic printing of cells with positive magnetophoresis requires cells (diamagnetic) to be labeled with magnetic nanoparticles to obtain a positive magnetic susceptibility. The labeled cells are then placed inside a culture well or vessel over a magnet arrangement. Presence of an inhomogeneous magnetic field concentrates magnetized cells into a desired geometry at regions of high magnetic field strength, resulting in the formation of a 3D cellular structure over time. (**b**) The cells are suspended within a Gadolinium-based paramagnetic salt solution prepared in cell media and then placed over a magnet arrangement. Magnetic printing of cells via negative magnetophoresis displaces diamagnetic cells to regions of low magnetic field strength, facilitated by the presence of paramagnetic salts. When sufficient cell–cell interactions have occurred, the inhomogeneous magnetic field and paramagnetic salt solution can be removed. The cell cluster continues to form cell–cell interactions and form a stable 3D cellular structure. Created in BioRender. Gupta, T. (2026) https://BioRender.com/fh0zi37 (access on 12 April 2026).

Magnetic printing is an emerging solution to engineer tissue constructs in situ [82,83]. Cell-based magnetic bioinks have been used to form sophisticated cell patterns and unique geometries such as thick 3D cell clusters [7], stacked co-cultures [8,84], rings [7,85], spheroids [86], and linear structures [65]. The formation of 3D cell clusters via positive magnetophoresis requires cells to internalize or be labeled with magnetic nanoparticles (MNPs), as described in Figure 2a. To improve biocompatibility and limit cytotoxic effects, MNPs are pre-coated with a biocompatible agent such as bovine serum albumin (BSA) [7]. The MNP-labeled cells attain positive magnetic susceptibility and move towards a region of high magnetic field strength, allowing their manipulation via action-from-a-distance with a static or dynamic external magnetic field. With intimate cell–cell contacts, cellular interactions produce ECM, which holds the structure together without an artificial scaffold [7].

Alternatively, magnetic cell-laden bioinks can exist as label-free cell suspensions in a magnetic cell culture medium (Figure 2b). For label-free magnetic printing, a magnetic bioink is prepared by suspending diamagnetic cells in a culture medium supplemented with a paramagnetic salt. In the presence of a magnetic field, the cells are displaced towards regions of lower magnetic field strength. Once the cells reach their equilibrium positions and synthesize sufficient ECM to hold their aggregated shape, the magnetic field is removed, and the paramagnetic medium is replaced with a regular culture medium. The assembled 3D cellular structure temporarily contracts due to intercellular interactions and, subsequently, grows through cell proliferation.

## 4. Label-Based Magnetic Cell Manipulation

A common label-based magnetic manipulation method tethers MNPs to cells. Commercially available MNPs consisting of iron oxides or nickel [87] are typically 1–100 nm in size and have high magnetic susceptibility that is conducive for manipulation [88,89]. MNPs can be synthesized bottom-up through Massart co-precipitation [88], oxidation of magnetic compounds [90] or biosynthesis [91], or top-down by grinding a bulk material [92]. The magnetization of these nanoparticles can be characterized by observing their magnetic hysteresis with a superconducting quantum interference device magnetometer [7]. MNPs can also be embedded into polymers to create magnetic aggregates (50–500 nm) or microspheres, also referred to as magnetic microbeads [73,74]. MNPs for clinical use must be biocompatible, have strong magnetic polarization, and maintain colloidal stability during application. To maintain colloidal stability, MNPs are coated with biocompatible surfactants to prevent their agglomeration, and subsequently, ligands can be bound to their coated surfaces for labeling or use in separation [41,73].

### 4.1. Single Cell Manipulation

Magnetic cell manipulation and separation capture target cells in situ using immunolabeled MNPs [73]. For clinical applications, cell separation isolates and identifies diseased cells in a suspension containing both healthy and diseased cells, such as circulating tumor cells (CTCs) during cancer diagnosis and prognosis. Since the number of CTCs can be small, e.g., 10 cells/mL of whole blood, sensitive and selective detection is required [72]. Alternatively, a quadrupole magnetic field can be employed for effective cell separation [93].

In a microfluidic device, magnetophoretic capture and particle trajectories of microspheres are dependent on the dipole strength of the field-inducing electromagnet, magnetic susceptibility of the particles, particle diameter, fluid viscosity, flow velocity, microchannel dimensions and distance of the dipole to the microchannel [94]. These parameters can be used to design microfluidic devices for the magnetic separation of cells and biological molecules using microelectro-mechanical systems for micro-total analysis systems [73]. For instance, micropatterned Ni-Co alloy permanent magnets were arranged underneath a glass microchannel to separate Michigan Cancer Foundation-7 (MCF-7) cells from whole blood [72].

Single cells can also be manipulated in a hybrid co-culture/scaffold environment to evaluate cell–cell and cell–ECM interactions. In an investigation, normal human dermal fibroblasts were either magnetically patterned with the magnetite-labeled MCF-10A/myr-Akt1 and MD Anderson-Metastatic Breast 231 (MDA-MB-231) cancer cells using a pin-holder device to observe cell–cell interactions, or introduced (non-patterned) within the collagen scaffold to study cell–ECM interactions [95]. This established a platform to observe complex tumor microenvironment (TME) interactions during in vitro screening.

### 4.2. Formation of 3D Cellular Structures and Tissues

During the label-based formation of 3D cellular structures and tissues, MNPs tether to cells or are internalized into the cells to enhance their magnetic susceptibility, allowing the cells to be manipulated in an inhomogeneous magnetic field gradient through action-from-a-distance. The NanoShuttle-PL^TM^ technology, consisting of gold nanoparticles, iron oxide, and poly-L-lysine, has been used with various cell lines to form in vitro models by non-specific labeling of the cell membrane. Examples include cultures of 3T3-L1, HEK 293 cells, co-cultures of human breast cancer cells with human fibroblast cell lines for the study of tumor-stroma interactions [1], 3T3 murine embryonic fibroblasts to demonstrate spheroid size as a metric for cytotoxicity [86], and human aortic smooth muscle cells (ASMC) formed in a ring structure to demonstrate functional vasoactivity [85].

Magnetic cell printing can be used in combination with artificial scaffolds or in a co-culture to replicate physiological phenomenon such as a heterogeneous TME [11,96]. Use of a scaffold provides the opportunity to observe the effects of paracrine signaling [11,97] and simulate tissue rigidity which orchestrates mechanosensing [98,99]. While a scaffold is necessary to model non-cellular components [96], and to mimic the TME, it also provides control over the conditions of the ECM [11] factors that influence tumor progression.

MNP chaperones can also be utilized to form tissue-like structures using spheroids as building blocks, as demonstrated by first forming spheroids composed of primary rat aortic smooth muscle cells (SMCs) and magnetoferritin, a less-toxic alternative to iron-based MNPs. These spheroids were then used to form a ring structure on the order of 10 mm within four days [100].

Label-based magnetic printing has revealed the resemblance of 3D cell clusters formed with internalized BSA-coated Fe_3_O_4_ particles in vivo tissues [7]. Independent cultures of human lung fibroblast (HFL-1) and human prostate cancer epithelial (PC-3) cells were arranged in various geometries to verify that the 3D cell clusters faithfully represent in vivo tissue using universal indicators of tissue formation: intercellular interactions and tissue morphology [7]. Morphologically, 3D cell clusters are more spheroidal than their 2D controls. The results verified that 3D magnetic printing enhanced intercellular interactions mediated by extracellular fibers, which is typical of tissues in vivo. The evolving morphology of a ring-like 3D cell cluster was also investigated. PC-3 and HFL-1 cells organized into a multi-layer sheet morphology while HFL-1 cells depicted spheroidal morphology. These arrangements are typical of epithelial and fibroblast morphologies in vivo, respectively.

Co-cultures have been used to form an aortic valve composed of valvular interstitial cells (VICs) and endothelial cells (VECs) extracted and isolated from a porcine heart [84]. NanoShuttle-PL^TM^ were used to levitate labeled cells at the air–liquid interface of the cell culture suspension, forming disk-like structures of each cell line within 4 h. These individual cell disks were then stacked on each other, with the VECs atop VICs. The interactions between the two disks took an additional 4 h, totaling 8 h of formation for the aortic valve co-culture (AVCC). Following three days of levitation, the AVCCs were harvested for phenotypic and functional biomarkers by immunohistochemistry staining and gene expression profiles, respectively. These miniaturized tissue constructs demonstrated the potential of magnetic levitational assays to form representative 3D tissue models. The AVCCs maintained the phenotype and functions of each individual cell line after co-culture formation and expressed relevant ECM markers. The rapid formation and relevant expressions observed in these AVCCs is encouraging for the potential of other tissue constructs for in vitro modeling of diseases.

A similar co-culture assembly process was used to form an airway-to-circulation facing bronchial wall using a magnetized Teflon pen that assembled monotypic 3D cultures into a stacked structure [8]. A stacked layer of epithelial cells, SMCs, pulmonary fibroblasts and pulmonary endothelial cells was used to create a bronchial co-culture model. Although use of these cell lines in a co-culture is not unique, this study demonstrated asymmetrical ECM formation (with emphasis on collagen type I), physiological phenotypic markers and cell morphologies for the first time.

The formation of 3D cellular structures is not limited to mammalian cells. The 3D magnetic printing of *E. coli* and *S. aureus* was facilitated by the internalization of sugar-coated MNPs to form spheroid, ring, and flat disk-like structures using N52 magnets [101]. Here, glucose, galactose, maltose, and sucrose-coated MNPs were shown to have no toxic effects on the bacteria cultures. The use of glucose-coated MNPs was shown to effectively modulate the swarming process of an *E. coli* liquid culture to promote biofilm formation and allow for subsequent studies.

## 5. Label-Free Magnetic Cell Manipulation

Label-free magnetic manipulation utilizes the intrinsic magnetic susceptibility of the material, where both positive and negative magnetophoresis are possible while manipulating paramagnetic and diamagnetic particles, respectively. For example, when whole blood is subjected to an external magnetic field, oxygenated red blood cells experience positive magnetophoresis while white blood cells experience negative magnetophoresis [102].

Since the magnetic susceptibility of diamagnetic materials is typically much weaker than paramagnetic materials, a diamagnetic material can be suspended in a paramagnetic solution, where the two can be separated by an applied magnetic field. The label-free manipulation of objects is also possible in non-magnetic mediums such as air, which can even lift small animals such as frogs and mice, although the magnetic strength required is not safe without protective equipment [103]. It is also possible to manipulate the orientation of diamagnetic objects by simply rotating an imposed dynamically changing magnetic field gradient [104].

### 5.1. Single Cell Manipulation

Single cell separation through negative magnetophoresis exploits the intrinsic diamagnetic properties of target cells in a heterogeneous cell suspension. The inherent differences in the densities of different cells or their status based on cellular events can be used to establish distinct levitational heights that serve as biophysical markers [105]. For clinical applications, label-free cell separation offers the ability to recirculate sorted cell fractions back into the body, a significant advantage over label-based efforts.

Cells can also be suspended in a paramagnetic medium to enhance label-free manipulation. Human histolytic lymphoma monocytes (U937) have been separated from red blood cells in a gadopentatic acid (Gd-DTPA)-based suspension contained in a microchannel [54]. Based on theoretical analysis, as the concentration of Gd-DTPA was increased from 0 to 80 mM, the separation efficiency also increased, in good agreement with experimental measurements [54].

Single living cells from a variety of organisms including mouse (NIH/3T3), yeast (*Saccharomyces cerevisiae*) and algae (*Chlamydomonas reinhardtii*) have been successfully levitated by suspending them in a solution of 40 mM Gd-DTPA [66]. Jurkat cells (human T lymphocytes) suspended in 5–10 mM of GdDO3A were levitated using a N52 micromagnet array [106], while *Saccharomyces cerevisiae* were levitated in 20 mM Gd-DTPA using a micromagnet array [67]. The positioning of the levitated cells followed the magnetic field gradient. These magnetic cell traps have the potential to fabricate biological microsystems using lab-on-chip devices [62].

Cells can also be suspended in a medium containing MNPs for label-free printing. For example, a ferrofluid containing BSA-coated iron oxide MNPs was used to form linear cellular chains of human umbilical vein endothelial cells (HUVEC) within minutes of applying a magnetic field [107]. This form of label-free cell manipulation with a ferrofluid utilizes the higher magnetic susceptibility of the ferrofluid in comparison to a paramagnetic salt solution and avoids the cytotoxicity associated with internalized or attached MNPs. The MNPs are not used as labels by either attachment or internalization. Instead, the dipoles of the ferrofluid particles shepherd the cells into linear chains. The morphology of the patterned linear chains was unchanged upon removal of the magnetic field or the ferrofluid. Culturing endothelial cells in linear chains produced capillary-like structures, a formidable challenge for tissue engineering.

### 5.2. Formation of 3D Cellular Structures and Tissues

The manipulation of single cells suspended in a paramagnetic medium can be extended to form 3D cellular structures [41]. A proof-of-concept method of 3D diamagnetophoretic cell printing used whole blood and a paramagnetic solution of Gd-DTPA in PBS [69]. WBCs and whole blood were examined separately for toxicity of Gd-DTPA. Various cell geometries were achieved by rearranging N52 magnets, where a line and three-pointed star were produced in circular vials. Simulations of the probable shape of the 3D cellular structure predicted equilibrium cell positions where the magnetic field strength was lowest. Similar to previous reports [108], circular cell clusters were formed on top of a magnet array but were contained in a rectangular vial with 1 mL of paramagnetic solution. Increasing the distance between the vial and the magnet array decreased the effective magnetic field strength, which increased the dimensions of the resulting clusters.

3D and 2D aggregates of MCF-7 and HUVEC, respectively, were assembled together on tissue culture-treated (TCT) surfaces, where these surfaces promoted cellular adhesion [70]. To produce co-cultures, HUVECs were first used to form a 2D monolayer in a regular culture medium without a magnetic field. Subsequent replacement of the medium with an MCF-7 magnetic bioink and use of magnet quartets facilitated the formation of a 3D cellular structure. Over time, cells on the periphery of the 3D cellular structure adhered to the TCT surface and interacted with the HUVECs to display a unique composite 2.5D morphology, namely, a 3D tumor adjacent to a 2D region containing endothelial cells that represents a metastatic tumor interacting with vasculature. The formation time for both 3D and 2.5D cell structures was 6 h. This rapid formation has not been observed with other methods without adding components to the culture medium to accelerate 3D cell structure formation. Both 3D and 2.5D cell structures formed through magnetic printing had a normal distribution of their maximum projected areas, confirming that the method is both rapid and highly reproducible.

**Figure 3:**
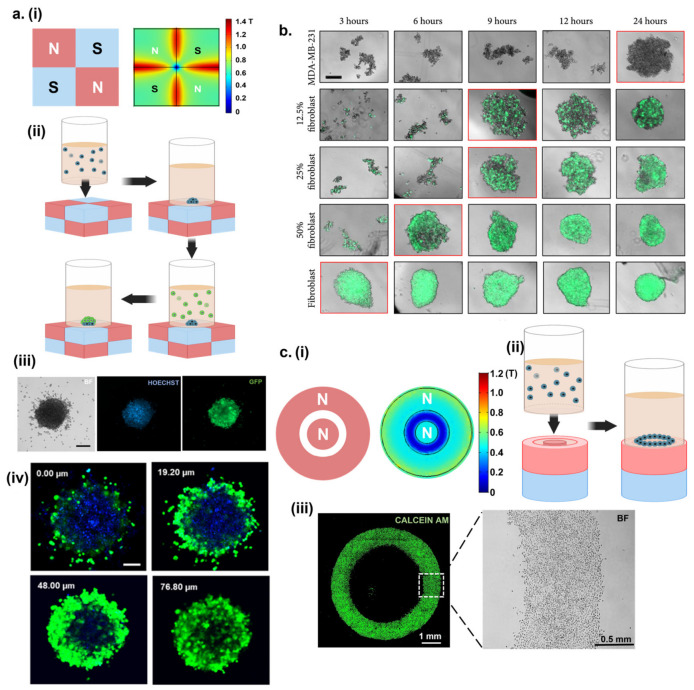
Label-free, contactless formation of 3D cellular structures formed from magnetic cell bioinks. (**a**) Magnetic flux density distribution at the surface of the magnet arrangement, experimental setups, and realization for a layer-on-layer structure formed from MCF-7 (core) and 3T3 cells (shell) on a hydrophobic plate using a magnet quartet in N-S-N-S orientation, resulting in a minimum magnetic flux density at their intersection (scale bar: 200 µm) [59]; (**b**) spherical 3D cellular structure composed of various ratios of MDA-MB-231 and 3T3 (EGFP) cells formed on an ultra-low attachment surface using four N52 4.5 × 4.5 × 4.5 mm magnets in N-S-N-S orientation, resulting in a minimum magnetic flux density at their intersection (scale bar: 100 µm) [56]; (**c**) Magnetic flux density distribution at the surface of the ring and cylinder magnet arrangement, experimental setups and realization for a annular monolayer of HBEC3 KT cells [55]. Panel (**a**,**c**) (**i**,**ii**) are Created in BioRender. Gupta, T. (2026) https://BioRender.com/eftwqvq (access on 12 April 2026).

Multi-layered 3D cellular structures have also been printed by diamagnetophoresis, where the first in situ proof-of-concept utilized an MCF-7 cell bioink to form a 3D cellular structure (Figure 3a) [59]. Subsequently, mouse embryonic fibroblast (3T3) cells were magnetically assembled to form a second layer, around and above the MCF-7 structure. This label-free cell assembly technique has the potential to produce tissue architectures such as skin, liver, and tubular structures such as the gastrointestinal tract.

3D co-cultures have also been formed using bioinks composed of 3T3 and breast cancer MDA-MB-231 cell bioinks (Figure 3b) [56]. With triple negative expression of estrogen, progesterone receptors, and human epidermal growth factor 2 (HER2), MDA-MB-231 is a clinically significant cell line to study cancer metastasis in vitro. However, this cell line is notoriously difficult to grow in 3D. For the first time, 3D MDA-MB-231 cellular structures were formed reproducibly by magnetic printing within 24 h without the use of additional reagents or an artificial scaffold, whereas 48 h were required for their formation under gravity. Instead of producing organized cell layers, MDA-MB-231 and 3T3 were mixed in different ratios to form co-culture aggregates. This study demonstrated the accelerated formation of 3D cellular structures and better reproducibility as the proportion of 3T3 fibroblasts was increased in the co-cultures. The investigation verified the conceptual use of fibroblasts as a glue to form clinically relevant co-culture models for disease modeling and personalized medicine.

Magnetic 3D printing is also enabling high-throughput therapeutic screening: a recent glioblastoma study used label-free magnetic assembly to form U-87 MG spheroids within 6 h and showed that combined 5-ALA and temozolomide produced the greatest sonodynamic antitumor effect, with a nearly additive enhancement in cell death relative to either sensitizer alone [14]. More broadly, recent sonodynamic therapy reviews identify 3D magnetically produced spheroid-based in vitro models important for improved sonosensitizer optimization to accelerate clinical translation of deep-tissue cancer therapies [109].

Single layered annular aggregates of human bronchial epithelial (HBEC 3KT) cells have been formed using a coaxially arranged cylinder and ring magnet on a tissue culture treated well plate for studying cell migration during various physiological processes. Effect of migration on a fibroblast-laden collagen hydrogel surface has also been studied. This work demonstrates the use of diamagnetophoresis in cell migration assay development that does not require surface scratch and offers flexibility in tuning the cell free area and its shape (Figure 3c).

Diamagnetic repulsion produces larger, multicellular constructs using prefabricated 3D cellular structures as building blocks. Bone marrow stem cell spheroids (D1 ORL UVA cell line) formed using a ring magnet were sequentially arranged into a cluster in a suspension of 200 mM Gd^3+^ [110]. As the number of spheroids increased, the area of the merged cluster also increased. In a supporting experiment, adipogenic-differentiated mouse osteoblasts (7F2 cell line) spheroids were formed in 100, 150, and 200 mM gadobutrol (Gadavist), a gadolinium-based contrast agent. As the concentration of Gd^3+^ decreased, the projected areas and perimeters of the structures increased [111]. This suggests that the magnetic strength of the medium and the number of building block units can be optimized to construct 3D clusters with varying densities and dimensions. The UVA and 7F2 cell lines had been used previously to produce 3D monotypic and layered co-culture using a microcapillary platform [77] and as monotypic cultures in a horizontal levitation system [112].

A functional tubular construct can be formed using a combination of magnetic levitation and acoustophoresis [2]. Here, tissue spheroids composed of human bladder smooth muscle cells (hbSMCs, myospheres) were produced using gravity in non-adherent microwells for one day. Following their assembly, the tissue spheroids were pooled together and suspended in a solution containing 20 mM gadobutrol. This suspension was contained in a cylindrical vessel within an acoustic apparatus, which was placed inside a Bitter electromagnet. The assembly process began by ‘turning on’ the magnetic field, which levitated the pooled spheroids in their culture vessel. To compensate for the low concentration of the paramagnetic salt, a high magnetic field intensity of 9.5 T was necessary. Once levitated, an acoustic radiation force was used to distribute the tissue spheroids vertically to form a solid tube. Both the external magnetic and acoustic body forces were held for 8 h to facilitate the fusion of the tissue spheroids into a tubular construct. To demonstrate functionality, the tubular hollow tissue-engineered structures were then exposed to 50 nM of endothelin-1, a vasoconstrictor peptide, for 2 h, which caused the tube to contract by 20% as compared to the control (no endothelin-1). The hybrid magnetoacoustic levitation technique allows rapid assembly of complex tissue architectures, a significant advantage over time-consuming traditional scaffold-based methods.

### 5.3. Magnetic Assembly Using Hydrogels

Magnetic printing can be extended to produce 3D cellular assemblies in hydrogels and biological scaffolds. UV-crosslinkable polyethylene glycol (PEG) hydrogels display paramagnetic behavior in a solution of PBS. As the UV exposure is increased to crosslink the PEG hydrogels, the gel magnetic susceptibility increases, enhancing their attraction toward a magnet [113].

Multistep mathematical and computational analyses have been performed to investigate the effects of magnetic susceptibility, hydrogel size, and distance from an external magnetic field. Levitation increased with increasing magnetic susceptibility and the size of paramagnetic gels. As the distance between the magnet and the hydrogels decreases, the magnetic force intensity acting on the hydrogels increases. The simulated motion of the paramagnetic hydrogel units is in good agreement with experimental observations. Hydrogel units of varying dimensions were arranged into linear shapes and 3D structures to demonstrate superior control and intentional organization in situ [113].

Bottom-up tissue engineering can also be accomplished using cell-laden hydrogels levitated in a paramagnetic medium. The levitation of hydrogels, including gelatin methacryloyl (GelMa) and polyethylene glycol dimethacrylate (PEGDMA), has been demonstrated to fabricate microgels into 3D, millimeter-scale assemblies [114]. The size of the 3D assembly decreased as a function of time and with increasing concentrations of Gd^3+^ and increased with an increasing number of microgels. The levitation height was shown to be a function of PEGDMA density, where gels with lower precursor compositions levitated at a higher equilibrium height.

The printing of 3D cellular structures is also possible within a hydrogel matrix. This was demonstrated by using polystyrene beads, followed by hollow poly(DL-lactide- co-glycolide) (PDLGA) drug delivery microcapsules and bovine mesenchymal stromal cells (MSCs) [115]. Here, methacrylated hyaluronic acid, a photo-crosslinkable hydrogel, was prepared with a solution of 200 mM gadodiamide and a photoinitator (0.5% *w*/*v* lithium phenyl-2,4,6-trimethylbenzoylphosphinate). Like magnetic printing in a fluid medium, diamagnetic objects were suspended in the solution and were guided to areas of the lowest magnetic field strength. Once the desired geometry had been achieved, the hydrogel was crosslinked to fix the initial positions of the cells. To reduce Gd toxicity, the crosslinked cell-laden hydrogels were washed to promote Gd release. The viability of the cells was not compromised during the six weeks of observation. In addition, the printed geometry exhibited depth-dependent cellularity similar to native cartilage tissue [115]. Table 2 summarizes key experimental studies using magnetic bioinks.

## 6. Concerns and Future Outlook

### 6.1. Nanoparticle Safety

The use of iron oxide nanoparticles is abundant in magnetic bioprinting. Iron is found naturally in red blood cells in the form of a heme molecule. Iron oxide nanoparticles are able to be metabolized by heme oxygenase-1 to form blood hemoglobin and hence maintain iron-cell homeostasis, and are generally considered biocompatible [118]. The cytotoxicity of iron oxide nanoparticles was evaluated for two human gliobastoma cell lines (T98G and U251), a human urinary bladder carcinoma cell line (ECV304), and a mouse fibroblast cell line (BALB/3T3) [119]. Here, magnetite nanoparticles were coated with rhamnose, a deoxy sugar, to induce cellular uptake and evaluated using various methods due to the lack of standardized procedures for nano-sized materials. The study reported that a concentration of 100 μg Fe/mL induced a cytotoxic response, resulting in an average of 35% cell death in the three cancerous cell lines due to mitochondrial damage. In contrast, no cytotoxicity was observed for the fibroblast cell line. For 3D magnetic printing via positive magnetophoresis, magnetoferritin (apoferritin-coated iron oxide nanoparticles) has been shown to improve cell viability compared to iron oxide MNPs and is considered to be a more biocompatible option. This is due to the activity of apoferritin that facilitates the oxidation of Fe(II) for subsequent iron storage by ferritin, an abundant protein found in our body that acts as an iron reserve [100].

### 6.2. Gadolinium Safety

Paramagnetic salts include various gadolinium-based agents, such as Gd-DTPA [56,58,59,69,70,120] and gadobutrol (Gd-BT-DO3A) [65,68], and manganese salts, such as manganese chloride (MnCl_2_) [121]. These agents must be selected after careful consideration of their toxic effects on cells. The structure, ionicity, stability, cell type and osmolality are some of the properties of various gadolinium-based salts that can affect the cellular uptake and thus the toxicity over long-term [122]. Cell viability of any cell type must be evaluated and the impact of Gd^3+^ ions on cellular pathways of interest must be considered in advance [123]. Due to the variations in reported findings, it is critical to consider Gd^3+^ safety for each individual cell type used, and to avoid making conclusions regarding safety and mechanism of action during exposure without performing thorough investigations. Short-term studies of three days on a monolayer of MCF-7 cells determined that exposure to 25 mM Gd-DTPA did not significantly reduce cell viability [58]. A similar evaluation of MDA-MB-231 and 3T3 cell monolayers established that 25 mM Gd-DTPA was safe for MDA-MB-231 and co-culture populations at 24 h of exposure. However, subsequent long-term studies over 2 weeks determined that Gd-DTPA inhibited the growth of the 3D cellular structures even though the exposure had been within pre-determined limits [56]. Therefore, it is not appropriate to assume long-term safety (more than 7 days) from short-term studies (24–72 h). In a separate investigation, Gd-DTPA has been shown to behave as a xenoestrogen when used with MCF-7, an estrogen receptor (ER) positive breast cancer cell line [124]. At low concentrations of Gd-DTPA (0.1 and 1 mM), proliferation and cell migration increased. This was not observed for the ER-negative cell line MDA-MB-231 and aneuploid mammary epithelial (Hs 578T) cells [124]. Moreover, exposure to high concentrations of gadolinium-based agents can affect intercellular interactions required for 3D cellular structure formation. For example, cell–substrate interactions of MCF-7 were significantly reduced when exposed to 50 mM of Gd-DTPA [58]. Furthermore, exposure to 250 mM Gd-BT-DO3A decreased the elastic modulus of the 3D cellular structures significantly [2]. The residual amount of Gadobutrol in HBEC3 KT cells after a wash was measured using inductively coupled plasma mass spectrometry and the residual Gd^3+^ ions did not affect the cell viability over 6 days [55]. Therefore, a couple of washes after the formation of desired cellular structure is recommended either immediately or over a day to remove any excess Gd^3+^ ions in the culture vessel for possible cellular uptake. Table 3 summarizes the cytotoxicity of various magnetic agents on different cell types. Although, a concentration range of 20–100 mM of the paramagnetic salt solution has been used for cell manipulation and cell aggregate printing using negative magnetophoresis, the impact of magnetic field gradient created by the permanent magnet and time of exposure need to be considered for long-term cell viability and effect.

Another important consideration is the effect of the osmotic pressures on cells in a suspension of a paramagnetic salt [35]. For reference, the osmotic pressure of a mammalian cell is 300 mOsm, while the osmotic pressure of a 40 mM solution of Gd-DTPA in Dulbecco’s Modified Eagle Medium (DMEM) is 278 mOsm [66]. As the gadolinium ion concentration in the surrounding medium increases, the cells expel water to reach equilibrium, which may lead to cell death. Therefore, lower paramagnetic salt concentration should be used to avoid cytotoxicity due to osmotic pressure differences. However, magnetic forces acting on the cells become weaker at lower paramagnetic salt concentrations, necessitating the use of stronger magnetic field gradients to achieve similar levitations. Besides cell viability, the long-term environmental impact of wastewater accumulation of gadolinium is not yet fully known or understood.

### 6.3. Biological Research Under Microgravity

Growing interest in space exploration and the possible inhabitation of other planets has motivated microgravity research related to long-term spaceflight. By subjecting 3D cellular constructs to the forces experienced during space travel, it is possible to mimic the biological effects of weightlessness. A particular interest is the study of weightlessness on the musculoskeletal system and bone density, demonstrated through studies using bioreactor cultures of chondrocytes on polyglycolic acid scaffolds [128] and porcine articular cartilage chondrocytes [129] in space. These early investigations determined that prolonged weightlessness experienced in microgravity environments compromises the mechanical stiffness of the engineered cartilage-cell constructs. These effects were not observed in microgravity simulations and normal gravity experiments on Earth [129].

Using magnetic levitation and printing of 3D tissue constructs in space [130], recent studies have focused on understanding the short- and long-term effects of weightlessness [65]. Such studies are rare since they are expensive, time-consuming, and require the cooperation of various space agencies and the training of astronauts to handle biological specimens. However, studies to determine the effect of weightlessness on 3D cellular assemblies are ongoing through various space missions.

### 6.4. Translational Outlook

The global 3D bioprinting market was valued at 3.07 B USD in 2025 and is expected to expand with a compound annual growth rate (CAGR) of 9.71% [131]. Although, magnetic bioink-based bioprinting is a fraction of this market size, it is expected to expand with a CAGR of 17% because it improves control, speed, and accuracy. The future of 3D cell culture is evident by the multitude of suppliers that provide reagents, cells, assays, equipment, and consumables for 3D cell culture. Bioinstrumentation companies (Luminex Corporations, Curiox Biosystems) offer magnetic bead-based immunoassay platforms and suppliers of magnetic beads used for cell manipulation and producing 3D structures (Thermofisher, Greiner Bio-One, Luna Nanotech, Creative Diagnostics, Bioclone Inc., and Sigma Aldrich). Greiner Bio-One offers a magnetic 3D bioassembler kit that uses magnetic beads to label cells and form 3D constructs using a magnetic assembler. Levitas Bio employs label-free magnetic manipulation to enrich and purify cells.

#### 6.4.1. Experimentally Validated Applications

The translational potential of magnetic bioinks lies in their ability to bridge fundamental biofabrication research with practical biomedical and industrial applications. By enabling rapid, scaffold-free, and spatially controlled assembly of living cells, magnetic bioinks can produce physiologically relevant 3D constructs that better approximate native tissue organization than conventional 2D culture systems. This capability is especially valuable for preclinical testing, where more representative in vitro models may improve prediction of therapeutic response, reduce dependence on animal experiments, and accelerate the evaluation of new drugs and biomaterials. The technology has already demonstrated utility in producing cardiac, vascular, airway, and tumor-related models, suggesting a path toward broader translational use in disease-specific platforms. Recent advancements include the formation of spheroids composed of neonatal primary rat cardiomyocytes using magnetic labels [132]. In addition to academic institutions, clinical research organizations (Crown Bioscience Inc., Aurelia Biosciences, NeOnc. Technology Holdings, Inc.) are adopting 3D cell culture testing methods. Recognition of the relevance of 3D cell models, along with the shortcomings of animal models, has caused the National Institute of Health to focus more on human-based research including organoids, tissue chips, microphysiological systems, AI, and mathematical methods [133]. This movement from the traditional clinical process is intended to improve translational success. However, widespread adoption within the industry is limited due to challenges in systematic assessments of 3D cultures, lack of standardization of assay protocols, limitations of imaging systems, and acceptance of the 3D cell culture data by regulatory agencies.

Regulatory approval of magnetic bioinks requires demonstrating that the materials are safe, reproducible, and capable of performing their intended biological function consistently. Developers must show that results can be repeated across different laboratories and manufacturing sites using standardized protocols, with consistent outcomes such as cell viability, magnetic responsiveness, print fidelity, and 3D tissue function. Regulatory agencies also require detailed characterization of the magnetic bioink composition, including nanoparticle or paramagnetic salt properties, sterility, stability, and batch-to-batch consistency under Good Manufacturing Practice (GMP) conditions. Extensive safety testing is needed to evaluate cytotoxicity, inflammation, immune responses, nanoparticle accumulation, toxicity associated with the paramagnetic salt, and long-term effects, especially for implanted applications.

In addition, magnetic bioink systems must meet key functional performance tests and demonstrate concordance with established methods. Critical milestones must be met that demonstrate that the engineered tissues or 3D cell models behave similarly to trusted standards such as conventional 3D cultures, histology, animal models, or clinical data. Functional testing may include tissue maturation, mechanical strength, drug response, or regenerative capability depending on the application. Regulators also expect validation of magnetic field parameters and analytical methods to ensure reliable and controlled performance. Together, these requirements help establish that magnetic bioinks are reproducible, clinically relevant, and safe for therapeutic or research use.

#### 6.4.2. Emerging Applications

Magnetic bioink-based approaches are also well positioned for patient-specific medicine. The ability to assemble multicellular spheroids, layered co-cultures, and tissue-like constructs with minimal physical contact offers a flexible platform for modeling disease using clinically relevant cell populations. In this context, magnetic bioprinting could support personalized diagnostics, treatment selection, and ex vivo assessment of drug sensitivity, particularly in oncology and regenerative medicine. Thus, beyond biomedical research, the impact of magnetic biofabrication may extend to sustainability-driven applications. Advances in 3D bioprinting have already stimulated interest in cultivated meat, plant-derived biomaterials, and biodegradable printed constructs. Recent advances in 3D bioprinting offer solutions to these concerns in the form of lab-grown meat [134] and the use of plant-derived materials for tissue engineering [135], and 3D-printed biodegradable patches [136]. Magnetic assembly methods may complement these efforts by improving the organization of living cellular components and enabling more controllable bottom-up fabrication strategies. In this way, the field intersects not only healthcare innovation, but also with emerging efforts to develop sustainable biomanufacturing technologies.

Future translation will therefore depend not only on improving magnetic bioinks themselves, but also on building robust validation frameworks around them. Standardization of materials, magnetic exposure conditions, toxicity testing, and performance metrics will be essential for reproducibility across laboratories and eventual clinical or industrial use. As these challenges are addressed, magnetic bioinks are likely to become increasingly important tools for drug discovery, biosensing, tissue engineering, and precision medicine, with the potential to reshape how complex living systems are fabricated and studied.

## 7. Conclusions

Magnetic bioinks have emerged as a versatile platform for the contactless manipulation and assembly of biological materials in three dimensions. By combining magnetic agents such as nanoparticles or paramagnetic salts with cells and other biological components, these systems enable precise and reproducible spatial organization through positive or negative magnetophoresis within few hours. This capability expands the conventional concept of bioprinting by allowing scaffold-free, contactless in situ formation of cellular constructs with reduced mechanical disturbance and improved control over architecture.

The studies reviewed here show that magnetic bioinks support a broad range of applications, from single-cell manipulation and separation to the fabrication of spheroids, layered co-cultures, tissue-like assemblies, and hydrogel-integrated constructs. These approaches have already demonstrated value in modeling tumor microenvironments, vascular and airway tissues, biosensing systems, and other physiologically relevant in vitro platforms. Their speed, flexibility, and action-at-a-distance operation make magnetic methods especially attractive for disease modeling, drug screening, and emerging biofabrication strategies.

At the same time, several challenges must be addressed before magnetic bioinks achieve wider translational use. The long-term effects of magnetic nanoparticles, the toxicity and osmotic consequences of paramagnetic salts, variability in magnetic field configurations, and limited standardization across studies remain important barriers. These issues highlight the next steps for research and the need for better material selection, reproducible fabrication protocols, and rigorous validation of biological outcomes over both short and extended culture periods.

Overall, magnetic bioinks represent a promising and rapidly developing direction in 3D bioprinting. Their ability to generate physiologically relevant cellular structures without conventional scaffolds positions them as powerful tools for biomedical research and future clinical innovation. As safety, reproducibility, and regulatory readiness improve, magnetic bioink-based biofabrication is likely to play an increasingly important role in personalized medicine, tissue engineering, and the development of advanced in vitro model systems.

## Figures and Tables

**Table 1:** Comparison of manipulation and 3D printing methods using magnetic bioink.

Method	Principle of Operation	Magnetic Agent	Typical Manipulation or Formation Time	Advantages	Limitations	Applications
Label-based single-cell manipulation	This method is based on positive magnetophoresis, which involves labeling specific cells with antibody-conjugated MNPs to sort or manipulate surface proteins using an external magnetic field.	Iron oxide MNPs, NanoShuttle-PL ^TM^, magnetoferritin	Few minutes to an hour	Inexpensive and easy to use compared to traditional methods like flow cytometry [41] Preserves cell viability [42] Compatible with microfluidic devices for precise cell manipulation	MNP internalization can interfere with cellular processes [41] Variable cellular uptake of MNPs can limit assay standardization [43]	Rare cell isolation [41] Cell sorting [44] Investigation of intracellular protein transport dynamics [45] Manipulation of mechanosensitive ion channels [46]
Label-based formation of 3D cellular structures	This bioprinting method uses magnetized cells or cell-embedded hydrogels functionalized with MNPs to produce 3D structures through positive magnetophoresis.		Initial clustering within 5 to 30 min Cohesive structure formation within 2–24 h	High-throughput and rapid assembly of cellular aggregates [47] Scalable fabrication of complex 3D multicellular structures [47,48]	MNPs can significantly affect stem cell functions, such as migration, in a concentration-dependent manner [49] Magnetic labeling can alter gene expression [49]	Spheroid formation and micropatterning [42] Fabrication of muscle constructs using MNP-incorporated cell-laden gels [50]
Label-free single-cell manipulation	This technique employs negative magnetophoresis, which leverages differences in magnetic susceptibility between cells and their surrounding medium to manipulate target cells based on their size or density.	Gd-DTPA, Gadobutrol, MnCl_2_	Few minutes to an hour	Facile and inexpensive method [51] Easy integration with microfluidic devices for rapid cell manipulation [52,53] High-throughput cell separation [53]	High magnetic field gradients are required for cell manipulation because of the small magnetic susceptibility differences between cells and the surrounding medium [54]	Density-based cancer cell sorting [52] Separation of blood cells and plasma [53]
Label-free formation of 3D cellular structures	This bioprinting method involves exposing diamagnetic cells suspended in a paramagnetic medium to an inhomogeneous magnetic field to form cellular aggregates through negative magnetophoresis.		Initial clustering within 15 min to an hour Cohesive structure formation within 2–6 h	Rapid cell assembly [51,55,56] Scalable fabrication of complex cellular structures [55,57]	Paramagnetic agents can be cytotoxic at high concentrations (>100 mM) [47] Cell types, such as neuronal cells, can uptake paramagnetic agents, potentially affecting their functionality [47]	Spheroid formation [58] Fabrication of layer-on-layer and annular cell aggregates [59] Formation of patterned cell clusters [57]

**Table 2:** Representative experimental studies for 3D printing using magnetic bioinks.

Cell Type	Magnet Configuration	Magnetic Agent	Culture Duration (Post Printing)	Cellular Structure	Primary Outcomes
Murine colon carcinoma (CT26) and human glioblastoma cells (U-87 MG) [116]	An array of cylindrical neodymium magnets (6 mm × 2 mm)	Maghemite (γ-Fe_2_O_3_) nanoparticles (1 mM)	5 days	Spheroids	The cell assembly method generated cohesive spheroids with high sphericity within 24 h Spheroid maturation influenced cell invasion and restricted drug penetrability
Aortic valvular interstitial cells (VICs) and endothelial cells (VECs) isolated from porcine hearts [84]	An array of 24 neodymium magnets (Bio-Assembler Kit, Nano3D Biosciences)	NanoShuttle-PL^TM^ (gold nanoparticles, iron oxide, and poly-L-lysine) (50 μL/mL medium)	3 days	Layered 3D aggregates	The magnetic technique preserved cellular phenotype and function in the aortic valve 3D co-cultures Gene expression analysis suggested that the VICs in the co-cultures were quiescent, which was attributed to the stiffness difference between 2D and 3D cultures.
Rat vascular smooth muscle cells (A10) and primary human aortic smooth muscle cells [85]	An array of 96 ring-shaped neodymium magnets (4.76 mm (OD) × 1.59 mm (ID))	NanoShuttle-PL^TM^ (1 μL per 10^4^ cells)	1 day	Ring-shaped aggregates	The magnetic technique assembled cellular rings with higher throughput than standard methods like wire myography The contractile behavior of the bioprinted rings was consistent with known vasoactive responses
Human bone marrow neuroblastoma cells (SH-SY5Y) [117]	MagLev system featuring two neodymium disk magnets arranged in an anti-Helmholtz configuration	Gadobutrol (10 mM)	9 days	Spheroids	The SH-SY5Y cells in the magnetically assembled spheroids successfully differentiated into a neuron-like phenotype in response to biochemical factors while maintaining high viability The differentiated SH-SY5Y spheroids exhibited neurotoxicity to A*β* 1–42 aggregates. This response was used to validate an Alzheimer’s disease model
Human breast cancer cells (MDA-MB-231) and murine fibroblasts (NIH/3T3) [56]	An array of neodymium cube magnets (4.5 mm × 4.5 mm × 4.5 mm) arranged in an N-S-N-S configuration	Gadolinium diethylenetriamine pentaacetic acid (25 mM)	14 days	Co-culture Spheroids	The cell assembly technique produced MDA-MB-231 3D aggregates within 24 h, significantly faster than the standard gravitational settling method Co-culturing MDA-MB-231 cells with fibroblasts accelerated the aggregate formation and enhanced reproducibility
Human bronchial epithelial cells (HBEC3 KT) [55]	An array of coaxially arranged neodymium ring magnets (12.7 mm (OD) × 6.35 mm (ID) × 6.35 mm) and cylinder magnets (3.175 mm (D) × 6.35 mm)	Gadobutrol (25 mM)	6 days	Ring-shaped aggregates	The magnetic exclusion technique produced ring-shaped cell aggregates reproducibly within 3 h The ring aggregates exhibited EGF concentration-dependent closure of cell-free areas, which was significantly greater on collagen-fibronectin-coated surfaces compared to tissue culture-treated surfaces
U-87 MG Glioblastoma [14]	An array of neodymium cube magnets (4.5 mm × 4.5 mm × 4.5 mm) arranged in an N-S-N-S configuration	Gadobutrol (25 mM)	5 days	Spheroids	Enhanced sonodynamic therapy screening with 5-ALA and TMZ sonosensitizers

**Table 3:** Safety and toxicity considerations for various magnetic agents used for 3D cell printing.

Magnetic Agent	Cell Type/Animal Model	Toxicity
Iron oxide nanoparticles (INPs; mean diameter = 6 ± 1.2 nm) and chitosan oligosaccharide-coated iron oxide nanoparticles (CSO-INPs; mean diameter = 8 ± 2.7 nm)	Human cervix carcinoma (HeLa), human lung carcinoma (A549), and human embryonic kidney (HEK 293) cells [125]	HeLa and A549 cells treated with INPs (4 μg/μL, 72 h) exhibited higher cell death (~50–60%) compared to cells treated with CSO-INPs TEM imaging revealed enhanced remodeling of the inner mitochondrial membrane and loss of mitochondrial membrane integrity in HeLa and A549 cells treated with INPs compared to CSO-INPs (4 μg/μL, 48 h treatment) Treatment with INPs caused higher depolarization of the mitochondrial membrane in HeLa, A549, and HEK 293 cells than with CSO-INPs (4 μg/μL, 48 h treatment)
Iron oxide nanoparticles (INPs) coated with glycol (PEG) (10 nm, SMG-10; 30 nm, SMG-30) and poly(ethylenimine) (PEI) (10 nm, SEI-10)	Murine macrophages (RAW264.7) and human ovarian cancer cells (SKOV3); BALB/c mice [126]	Higher cellular uptake of SEI-10 was observed in RAW264.7 and SKOV3 cells compared to SMG-10 and SMG-30 after 4 h of incubation Compared to SMG-10 and SMG-30, SEI-10 induced significant concentration-dependent apoptosis in SKOV-3 and RAW264.7 cells after 24 h of incubation BALB/c mice intravenously injected with INPs (1.5 mg/kg) showed normal hepatic and renal function at 2 weeks post-injection. However, significant animal deaths occurred at higher doses of SEI-10 (2.5 and 5 mg/kg) compared to SMG-10 and SMG-30 within 24 h post-injection
ε-Poly-L-lysine (PLL)-modified magnetic nanoparticles (hydrodynamic diameter = 253 ± 10 nm)	Human umbilical cord-derived mesenchymal stem cells (MSCs); Astrocyte cultures prepared from spinal cord tissues of neonatal C57BL/6 mice [127]	No significant changes in MSC morphology, cell viability, or differentiation potential were observed due to the uptake of PLL-MNPs (0.1 μg/μL) No significant changes in MSC proliferation were observed after PLL-MNP treatment at 48 h Wound healing assay revealed enhanced migration of PLL-MNP-labeled MSCs compared to the unlabeled control at 12 h Secretion of IL-10, IL-13, RANTES, and VEGF was significantly upregulated in MNP-labeled cells at 24 h MNP-labeled MSCs promoted the transition of activated astrocytes to an anti-inflammatory phenotype
Paramagnetic agents Gadobutrol (Gx), Omniscan/Gadodiamide (Ox) and Dotarem/Gadoteric acid (Dx) (0, 30, 50, 100, and 200 mM)	Murine fibroblasts (NIH/3T3) and human non-small-cell lung cancer (HCC827) cells [39]	Among the paramagnetic agents, Gx was the most biocompatible. At 30 mM, ~90–95% viability was observed for Gx with no significant change through Day 5. However, viability decreased to ~50–55% on Day 7. In contrast, Dx (30 mM) was the most cytotoxic, with ~50% viability on Day 1 All paramagnetic agents exhibited concentration-dependent cytotoxicity. At 200 mM, Gx-treated cells had the highest viability (~30%) on Day 1.
Paramagnetic agents Gadobutrol (Gd-BT-DO3A), Magnevist (Gd-DTPA), Omniscan (Gd-DTPA-BMA), Dotarem (Gd-DOTA), and Multihance (Gd-BOPTA) (0, 10, 25, 50, 100, and 200 mM)	Murine bone marrow (D1 ORL UVA) and human breast cancer (MDA-MB-231) cells [65]	At 200 mM, Gd-DTPA, Gd-DTPA-BMA, Gd-DOTA, and Gd-BOPTA caused significant cell death after 72 h of incubation, while Gd-BT-DO3A only inhibited cell growth For other concentrations, Gd-BT-DO3A and Gd-DOTA demonstrated higher cell viability than the other agents Gd-DTPA-BMA suppressed cell growth even at 25 mM
Gadolinium diethylenetriamine pentaacetic acid (Magnevist; Gd-DTPA) (0, 1, 10, 25, 50, 75, 100, and 125 mM)	Human breast cancer (MCF-7) cells [58]	No significant decrease in cell viability was observed for all Gd-DTPA concentrations within 24 h of exposure. However, cell viability decreased significantly on subsequent days in a concentration-dependent manner, with ~10–20% viability observed for 50–125 mM Gd-DTPA at 72 h

## Data Availability

No new data was generated or analyzed in this review. Thus, data sharing is not applicable.

## Abbreviations

The following abbreviations are used in this manuscript:

3D	Three-dimensional
2D	Two-dimensional
ECM	Extracellular matrix
**B**	Total magnetic field
**M**	Magnetization
**H**	Applied magnetic field
*μ*_0_	Permeability of vacuum
*χ*	Magnetic susceptibility
**F**	Magnetic body force
N	Demagnetization factor
a	Particle radius
MNP	Magnetic nanoparticle
BSA	Bovine serum albumin
CTC	Circulating tumor cells
MCF-7	Michigan Cancer Foundaiton-7
MDA-MB-231	MD Anderson-Metastatic Breast 231
TME	Tumor microenvironment
HEK 293	Human embryonic kidney 293
SMC	Smooth muscle cells
HFL-1	Human lung fibroblast
PC-3	Human prostate cancer epithelial cells
VIC	Valvular interstitial cells
VEC	Valvular endothelial cells
AVCC	Aortic valve co-culture
HUVEC	Human umbilical vein endothelial cells
U937	Human histolytic lymphoma monocytes
Gd-DTPA	Gadopentatic acid
TCT	Tissue culture treated
HBEC	Human bronchial epithelial cells
HER2	Human epidermal growth factor 2
PEG	Polyethylene glycol
GelMa	Gelatin methacryloyl
PEGDMA	Polyethylene glycol dimethacrylate
PDLGA	Poly(DL-lactide- co-glycolide)
MSC	Mesenchymal stromal cells

## References

[1] Jaganathan H., Gage J., Leonard F., Srinivasan S., Souza G.R., Dave B., Godin B. (2014). Three-dimensional in vitro co-culture model of breast tumor using magnetic levitation. Sci. Rep..

[2] Parfenov V.A., Koudan E.V., Krokhmal A.A., Annenkova E.A., Petrov S.V., Pereira F.D., Karalkin P.A., Nezhurina E.K., Gryadunova A.A., Bulanova E.A. (2020). Biofabrication of a Functional Tubular Construct from Tissue Spheroids Using Magnetoacoustic Levitational Directed Assembly. Adv. Healthc. Mater..

[3] Charbe N., McCarron P.A., Tambuwala M.M. (2017). Three-dimensional bio-printing: A new frontier in oncology research. World J. Clin. Oncol..

[4] Cooper M., Tanaka M., Puri I. (2010). Coupled mathematical model of tumorigenesis and angiogenesis in vascular tumours. Cell Prolif..

[5] Ganguly R., Puri I. (2007). Mathematical model for chemotherapeutic drug efficacy in arresting tumour growth based on the cancer stem cell hypothesis. Cell Prolif..

[6] Kenny P.A., Lee G.Y., Myers C.A., Neve R.M., Semeiks J.R., Spellman P.T., Lorenz K., Lee E.H., Barcellos-Hoff M.H., Petersen O.W. (2007). The morphologies of breast cancer cell lines in three-dimensional assays correlate with their profiles of gene expression. Mol. Oncol..

[7] Ghosh S., Kumar S., Puri I., Elankumaran S. (2016). Magnetic assembly of 3D cell clusters: Visualizing the formation of an engineered tissue. Cell Prolif..

[8] Tseng H., Gage J.A., Raphael R.M., Moore R.H., Killian T.C., Grande-Allen K.J., Souza G.R. (2013). Assembly of a three-dimensional multitype bronchiole coculture model using magnetic levitation. Tissue Eng. Part C Methods.

[9] Costa E.C., Moreira A.F., de Melo-Diogo D., Gaspar V.M., Carvalho M.P., Correia I.J. (2016). 3D tumor spheroids: An overview on the tools and techniques used for their analysis. Biotechnol. Adv..

[10] Kunz-Schughart L.A., Freyer J.P., Hofstaedter F., Ebner R. (2004). The use of 3-D cultures for high-throughput screening: The multicellular spheroid model. J. Biomol. Screen..

[11] Xin X., Yang H., Zhang F., Yang S.-T. (2019). 3D cell coculture tumor model: A promising approach for future cancer drug discovery. Process Biochem..

[12] Brancato V., Oliveira J.M., Correlo V.M., Reis R.L., Kundu S.C. (2020). Could 3D models of cancer enhance drug screening?. Biomaterials.

[13] Zanoni M., Piccinini F., Arienti C., Zamagni A., Santi S., Polico R., Bevilacqua A., Tesei A. (2016). 3D tumor spheroid models for in vitro therapeutic screening: A systematic approach to enhance the biological relevance of data obtained. Sci. Rep..

[14] Datta P., Lee N.S., Moolayadukkam S., Sahu R.P., Yu X., Guo T., Zhou Q., Wang Y., Puri I.K. (2024). In Vitro Sonodynamic Therapy Using a High Throughput 3D Glioblastoma Spheroid Model with 5-ALA and TMZ Sonosensitizers. Adv. Healthc. Mater..

[15] Clevers H. (2016). Modeling development and disease with organoids. Cell.

[16] Schutgens F., Clevers H. (2020). Human organoids: Tools for understanding biology and treating diseases. Annu. Rev. Pathol. Mech. Dis..

[17] Soto F., Guimarães C.F., Reis R.L., Franco W., Rizvi I., Demirci U. (2021). Emerging biofabrication approaches for gastrointestinal organoids towards patient specific cancer models. Cancer Lett..

[18] Bhise N.S., Manoharan V., Massa S., Tamayol A., Ghaderi M., Miscuglio M., Lang Q., Zhang Y.S., Shin S.R., Calzone G. (2016). A liver-on-a-chip platform with bioprinted hepatic spheroids. Biofabrication.

[19] Essaouiba A., Jellali R., Shinohara M., Scheidecker B., Legallais C., Sakai Y., Leclerc E. (2021). Analysis of the behavior of 2D monolayers and 3D spheroid human pancreatic beta cells derived from induced pluripotent stem cells in a microfluidic environment. J. Biotechnol..

[20] Tsai H.-F., Trubelja A., Shen A.Q., Bao G. (2017). Tumour-on-a-chip: Microfluidic models of tumour morphology, growth and microenvironment. J. R. Soc. Interface.

[21] Ustun M., Rahmani Dabbagh S., Ilci I.S., Bagci-Onder T., Tasoglu S. (2021). Glioma-on-a-Chip Models. Micromachines.

[22] Langer E.M., Allen-Petersen B.L., King S.M., Kendsersky N.D., Turnidge M.A., Kuziel G.M., Riggers R., Samatham R., Amery T.S., Jacques S.L. (2019). Modeling tumor phenotypes in vitro with three-dimensional bioprinting. Cell Rep..

[23] Jakus A.E., Rutz A.L., Jordan S.W., Kannan A., Mitchell S.M., Yun C., Koube K.D., Yoo S.C., Whiteley H.E., Richter C.-P. (2016). Hyperelastic “bone”: A highly versatile, growth factor–free, osteoregenerative, scalable, and surgically friendly biomaterial. Sci. Transl. Med..

[24] Wan Z., Zhang P., Liu Y., Lv L., Zhou Y. (2020). Four-dimensional bioprinting: Current developments and applications in bone tissue engineering. Acta Biomater..

[25] Gao B., Yang Q., Zhao X., Jin G., Ma Y., Xu F. (2016). 4D bioprinting for biomedical applications. Trends Biotechnol..

[26] Madhuri D., Ozbolat I.T. (2020). 3D bioprinting of cells, tissues and organs. Sci. Rep..

[27] Gupta T., Ghosh R., Ganguly R. (2018). Acoustophoretic separation of infected erythrocytes from blood plasma in a microfluidic platform using biofunctionalized, matched-impedance layers. Int. J. Numer. Methods Biomed. Eng..

[28] Kang B., Shin J., Park H.-J., Rhyou C., Kang D., Lee S.-J., Yoon Y.-s., Cho S.-W., Lee H. (2018). High-resolution acoustophoretic 3D cell patterning to construct functional collateral cylindroids for ischemia therapy. Nat. Commun..

[29] Fan R., Piou M., Darling E., Cormier D., Sun J., Wan J. (2016). Bio-printing cell-laden Matrigel–agarose constructs. J. Biomater. Appl..

[30] Huang H., Qi X., Chen Y., Wu Z. (2019). Thermo-sensitive hydrogels for delivering biotherapeutic molecules: A review. Saudi Pharm. J..

[31] Knowlton S., Yenilmez B., Anand S., Tasoglu S. (2017). Photocrosslinking-based bioprinting: Examining crosslinking schemes. Bioprinting.

[32] Benetti E.M., Gunnewiek M.K., van Blitterswijk C.A., Vancso G.J., Moroni L. (2016). Mimicking natural cell environments: Design, fabrication and application of bio-chemical gradients on polymeric biomaterial substrates. J. Mater. Chem. B.

[33] Shahin-Shamsabadi A., Selvaganapathy P. (2020). Tissue-in-a-Tube: Three-dimensional in vitro tissue constructs with integrated multimodal environmental stimulation. Mater. Today Bio.

[34] Gao Q.-H., Zhang W.-M., Zou H.-X., Li W.-B., Yan H., Peng Z.-K., Meng G. (2019). Label-free manipulation via the magneto-Archimedes effect: Fundamentals, methodology and applications. Mater. Horiz..

[35] Tasoglu S., Yu C.H., Liaudanskaya V., Guven S., Migliaresi C., Demirci U. (2015). Magnetic levitational assembly for living material fabrication. Adv. Healthc. Mater..

[36] Ashammakhi N., Ahadian S., Zengjie F., Suthiwanich K., Lorestani F., Orive G., Ostrovidov S., Khademhosseini A. (2018). Advances and future perspectives in 4D bioprinting. Biotechnol. J..

[37] Ozefe F., Yildiz A.A. (2020). Magnetic Levitation Based Applications in Bioscience. Magnetic Levitation.

[38] Adhikari J., Roy A., Das A., Ghosh M., Thomas S., Sinha A., Kim J., Saha P. (2021). Effects of processing parameters of 3D bioprinting on the cellular activity of bioinks. Macromol. Biosci..

[39] Türker E., Demirçak N., Arslan-Yildiz A. (2018). Scaffold-free three-dimensional cell culturing using magnetic levitation. Biomater. Sci..

[40] Onbas R., Arslan Yildiz A. (2021). Fabrication of tunable 3D cellular structures in high volume using magnetic levitation guided assembly. ACS Appl. Bio Mater..

[41] Yaman S., Anil-Inevi M., Ozcivici E., Tekin H.C. (2018). Magnetic force-based microfluidic techniques for cellular and tissue bioengineering. Front. Bioeng. Biotechnol..

[42] Kappes M., Friedrich B., Pfister F., Huber C., Friedrich R.P., Stein R., Braun C., Band J., Schreiber E., Alexiou C. (2022). Superparamagnetic iron oxide nanoparticles for targeted cell seeding: Magnetic patterning and magnetic 3D cell culture. Adv. Funct. Mater..

[43] Zhao W., Cheng R., Miller J.R., Mao L. (2016). Label-free microfluidic manipulation of particles and cells in magnetic liquids. Adv. Funct. Mater..

[44] Bshara-Corson S., Burwell A., Tiemann T., Murray C. (2024). Digital Magnetic Sorting for Fractionating Cell Populations with Variable Antigen Expression in Cell Therapy Process Development. Magnetochemistry.

[45] Landis M.K., Kunze A. (2026). Cell-Internal Nanomagnetic Forces Alter Cytoskeletal Protein Transport Dynamics in Developing Excitatory Axons. Nano Lett..

[46] Del Sol-Fernández S., De Simone M., Fernández-Afonso Y., Garcia-Gonzalez D., Martínez-Vicente P., Van Zanten T., Fratila R.M., Moros M. (2026). MagPiezo: A magnetogenetic platform for remote activation of endogenous Piezo1 channels in endothelial cells. Adv. Funct. Mater..

[47] Hu H., Krishaa L., Fong E.L.S. (2023). Magnetic force-based cell manipulation for in vitro tissue engineering. APL Bioeng..

[48] Jafari J., Han X.-l., Palmer J., Tran P.A., O’Connor A.J. (2019). Remote control in formation of 3D multicellular assemblies using magnetic forces. ACS Biomater. Sci. Eng..

[49] Bulte J.W., Wang C., Shakeri-Zadeh A. (2022). In vivo cellular magnetic imaging: Labeled versus unlabeled cells. Adv. Funct. Mater..

[50] Hwangbo H., Chae S., Ryu D., Kim G. (2025). In situ magnetic-field-assisted bioprinting process using magnetorheological bioink to obtain engineered muscle constructs. Bioact. Mater..

[51] Cagan-Algan M., Anil-Inevi M., Kecili S., Inal E., Tekin H.C., Mese G., Ozcivici E. (2025). Negative magnetophoresis guided unidirectional cell patterning on culture surface. Biomed. Eng. Adv..

[52] Kecili S., Yilmaz E., Ozcelik O.S., Anil-Inevi M., Gunyuz Z.E., Yalcin-Ozuysal O., Ozcivici E., Tekin H.C. (2023). μDACS platform: A hybrid microfluidic platform using magnetic levitation technique and integrating magnetic, gravitational, and drag forces for density-based rare cancer cell sorting. Biosens. Bioelectron. X.

[53] Zeng L., Liu C., Yang Y., Hu S., Li R., Tan X., Shen J., Zhang Y., Huang S., Yang H. (2024). Power-free plasma separation based on negative magnetophoresis for rapid biochemical analysis. Microsyst. Nanoeng..

[54] Shen F., Hwang H., Hahn Y.K., Park J.-K. (2012). Label-free cell separation using a tunable magnetophoretic repulsion force. Anal. Chem..

[55] Gupta T., Gupta R., Dabaghi M., Sahu R.P., Hirota J.A., Puri I.K. (2021). Label-Free Cell Migration Assay Using Magnetic Exclusion. Adv. Mater. Technol..

[56] Mishriki S., Aithal S., Gupta T., Sahu R.P., Geng F., Puri I.K. (2020). Fibroblasts Accelerate Formation and Improve Reproducibility of 3D Cellular Structures Printed with Magnetic Assistance. Research.

[57] Ren T., Maitusong M., Zhou X., Hong X., Cheng S., Lin Y., Xue J., Xu D., Chen J., Qian Y. (2023). Programing cell assembly via ink-free, label-free magneto-archimedes based strategy. ACS Nano.

[58] Mishriki S., Abdel Fattah A.R., Kammann T., Sahu R., Geng F., Puri I. (2019). Rapid magnetic 3D printing of cellular structures with MCF-7 cell inks. Research.

[59] Gupta T., Aithal S., Mishriki S., Sahu R.P., Geng F., Puri I.K. (2020). Label-Free Magnetic-Field-Assisted Assembly of Layer-on-Layer Cellular Structures. ACS Biomater. Sci. Eng..

[60] Griffiths D.J. (2005). Introduction to Electrodynamics.

[61] Grob D.T., Wise N., Oduwole O., Sheard S. (2018). Magnetic susceptibility characterisation of superparamagnetic microspheres. J. Magn. Magn. Mater..

[62] Iacovacci V., Lucarini G., Ricotti L., Menciassi A. (2016). Magnetic field-based technologies for lab-on-a-chip applications. Lab-on-a-Chip Fabrication and Application.

[63] Patton J.A. (1994). MR imaging instrumentation and image artifacts. Radiographics.

[64] Ghosh D., Gupta T., Sahu R.P., Das P.K., Puri I.K. (2020). Three-dimensional printing of diamagnetic microparticles in paramagnetic and diamagnetic media. Phys. Fluids.

[65] Anil-Inevi M., Yaman S., Yildiz A.A., Mese G., Yalcin-Ozuysal O., Tekin H.C., Ozcivici E. (2018). Biofabrication of in situ self assembled 3D cell cultures in a weightlessness environment generated using magnetic levitation. Sci. Rep..

[66] Winkleman A., Gudiksen K.L., Ryan D., Whitesides G.M., Greenfield D., Prentiss M. (2004). A magnetic trap for living cells suspended in a paramagnetic buffer. Appl. Phys. Lett..

[67] Frenea-Robin M., Chetouani H., Haddour N., Rostaing H., Laforet J., Reyne G. (2008). Contactless diamagnetic trapping of living cells onto a micromagnet array. Proceedings of the 2008 30th Annual International Conference of the IEEE Engineering in Medicine and Biology Society.

[68] Yaman S., Tekin H.C. (2020). Magnetic susceptibility-based protein detection using magnetic levitation. Anal. Chem..

[69] Abdel Fattah A.R., Meleca E., Mishriki S., Lelic A., Geng F., Sahu R.P., Ghosh S., Puri I.K. (2016). In Situ 3D Label-Free Contactless Bioprinting of Cells Through Diamagnetophoresis. ACS Biomater. Sci. Eng..

[70] Fattah A.R.A., Mishriki S., Kammann T., Sahu R.P., Geng F., Puri I.K. (2018). 3D cellular structures and co-cultures formed through the contactless magnetic manipulation of cells on adherent surfaces. Biomater. Sci..

[71] Ganguly R., Gaind A.P., Sen S., Puri I.K. (2005). Analyzing ferrofluid transport for magnetic drug targeting. J. Magn. Magn. Mater..

[72] Park J.W., Lee N.R., Cho S.M., Jung M.Y., Ihm C., Lee D.S. (2015). Microdevice for separation of circulating tumor cells using embedded magnetophoresis with V-shaped Ni-Co nanowires and immuno-nanomagnetic beads. Etri J..

[73] Ganguly R., Puri I.K. (2010). Microfluidic transport in magnetic MEMS and bioMEMS. Wiley Interdiscip. Rev. Nanomed. Nanobiotechnol..

[74] Ganguly R., Puri I.K. (2007). Field-assisted self-assembly of superparamagnetic nanoparticles for biomedical, MEMS and BioMEMS applications. Adv. Appl. Mech..

[75] Zahn M. (1979). Electromagnetic Field Theory: A Problem Solving Approach.

[76] Smistrup K., Hansen O., Bruus H., Hansen M.F. (2005). Magnetic separation in microfluidic systems using microfabricated electromagnets—Experiments and simulations. J. Magn. Magn. Mater..

[77] Sarigil O., Anil-Inevi M., Firatligil-Yildirir B., Unal Y.C., Yalcin-Ozuysal O., Mese G., Tekin H.C., Ozcivici E. (2021). Scaffold-free biofabrication of adipocyte structures with magnetic levitation. Biotechnol. Bioeng..

[78] Mironov V., Visconti R.P., Kasyanov V., Forgacs G., Drake C.J., Markwald R.R. (2009). Organ printing: Tissue spheroids as building blocks. Biomaterials.

[79] Laschke M.W., Menger M.D. (2017). Life is 3D: Boosting spheroid function for tissue engineering. Trends Biotechnol..

[80] Ozler S.B., Kucukgul C., Koc B. (2015). Bioprinting with live cells. Bioprinting in Regenerative Medicine.

[81] Prendergast M.E., Solorzano R.D., Cabrera D. (2017). Bioinks for biofabrication: Current state and future perspectives. J. 3D Print. Med..

[82] Parfenov V.A., Mironov V.A., van Kampen K.A., Karalkin P.A., Koudan E.V., Pereira F.D., Petrov S.V., Nezhurina E.K., Petrov O.F., Myasnikov M. (2020). Scaffold-free and label-free biofabrication technology using levitational assembly in high magnetic field. Biofabrication.

[83] Parfenov V.A., Koudan E.V., Bulanova E.A., Karalkin P.A., Pereira F.D., Norkin N.E., Knyazeva A.D., Gryadunova A.A., Petrov O.F., Vasiliev M.M. (2018). Scaffold-free, label-free and nozzle-free biofabrication technology using magnetic levitational assembly. Biofabrication.

[84] Tseng H., Balaoing L.R., Grigoryan B., Raphael R.M., Killian T., Souza G.R., Grande-Allen K.J. (2014). A three-dimensional co-culture model of the aortic valve using magnetic levitation. Acta Biomater..

[85] Tseng H., Gage J.A., Haisler W.L., Neeley S.K., Shen T., Hebel C., Barthlow H.G., Wagoner M., Souza G.R. (2016). A high-throughput in vitro ring assay for vasoactivity using magnetic 3D bioprinting. Sci. Rep..

[86] Tseng H., Gage J.A., Shen T., Haisler W.L., Neeley S.K., Shiao S., Chen J., Desai P.K., Liao A., Hebel C. (2015). A spheroid toxicity assay using magnetic 3D bioprinting and real-time mobile device-based imaging. Sci. Rep..

[87] Abdel Fattah A.R., Majdi T., Abdalla A.M., Ghosh S., Puri I.K. (2016). Nickel nanoparticles entangled in carbon nanotubes: Novel ink for nanotube printing. ACS Appl. Mater. Interfaces.

[88] Fattah A.R.A., Ghosh S., Puri I.K. (2016). Printing microstructures in a polymer matrix using a ferrofluid droplet. J. Magn. Magn. Mater..

[89] Abdel Fattah A.R., Ghosh S., Puri I.K. (2016). Printing three-dimensional heterogeneities in the elastic modulus of an elastomeric matrix. ACS Appl. Mater. Interfaces.

[90] Gilchrist R., Medal R., Shorey W.D., Hanselman R.C., Parrott J.C., Taylor C.B. (1957). Selective inductive heating of lymph nodes. Ann. Surg..

[91] Schröfel A., Kratošová G., Šafařík I., Šafaříková M., Raška I., Shor L.M. (2014). Applications of biosynthesized metallic nanoparticles–a review. Acta Biomater..

[92] Shabatina T.I., Vernaya O.I., Shabatin V.P., Melnikov M.Y. (2020). Magnetic Nanoparticles for Biomedical Purposes: Modern Trends and Prospects. Magnetochemistry.

[93] Zborowski M., Sun L., Moore L.R., Williams P.S., Chalmers J.J. (1999). Continuous cell separation using novel magnetic quadrupole flow sorter. J. Magn. Magn. Mater..

[94] Nandy K., Chaudhuri S., Ganguly R., Puri I.K. (2008). Analytical model for the magnetophoretic capture of magnetic microspheres in microfluidic devices. J. Magn. Magn. Mater..

[95] Okochi M., Matsumura T., Honda H. (2013). Magnetic force-based cell patterning for evaluation of the effect of stromal fibroblasts on invasive capacity in 3Dcultures. Biosens. Bioelectron..

[96] Kaemmerer E., Garzon T.E.R., Lock A.M., Lovitt C.J., Avery V.M. (2016). Innovative in vitro models for breast cancer drug discovery. Drug Discov. Today Dis. Models.

[97] Sung K.E., Su X., Berthier E., Pehlke C., Friedl A., Beebe D.J. (2013). Understanding the impact of 2D and 3D fibroblast cultures on in vitro breast cancer models. PLoS ONE.

[98] Wei S.C., Yang J. (2016). Forcing through tumor metastasis: The interplay between tissue rigidity and epithelial–mesenchymal transition. Trends Cell Biol..

[99] Chen W., Park S., Patel C., Bai Y., Henary K., Raha A., Mohammadi S., You L., Geng F. (2021). The migration of metastatic breast cancer cells is regulated by matrix stiffness via YAP signalling. Heliyon.

[100] Mattix B., Olsen T.R., Gu Y., Casco M., Herbst A., Simionescu D.T., Visconti R.P., Kornev K.G., Alexis F. (2014). Biological magnetic cellular spheroids as building blocks for tissue engineering. Acta Biomater..

[101] Neto L.A.A., Pereira T.M., Silva L.P. (2020). Magnetic nanoparticles coated with carbohydrates for 3D culture of bacteria. Mater. Sci. Eng. C.

[102] Fattah A.R.A., Ghosh S., Puri I.K. (2016). High gradient magnetic field microstructures for magnetophoretic cell separation. J. Chromatogr. B.

[103] Berry M.V., Geim A.K. (1997). Of flying frogs and levitrons. Eur. J. Phys..

[104] Subramaniam A.B., Yang D., Yu H.-D., Nemiroski A., Tricard S., Ellerbee A.K., Soh S., Whitesides G.M. (2014). Noncontact orientation of objects in three-dimensional space using magnetic levitation. Proc. Natl. Acad. Sci. USA.

[105] Durmus N.G., Tekin H.C., Guven S., Sridhar K., Yildiz A.A., Calibasi G., Ghiran I., Davis R.W., Steinmetz L.M., Demirci U. (2015). Magnetic levitation of single cells. Proc. Natl. Acad. Sci. USA.

[106] Kauffmann P., Dempsey N., O’Brien D., Combe S., Schaack B., Haguet V., Reyne G. Diamagnetic trapping of cells above micro-magnets. Proceedings of the 14th Biennial IEEE Conference on Electromagnetic Field Computation (CEFC).

[107] Krebs M.D., Erb R.M., Yellen B.B., Samanta B., Bajaj A., Rotello V.M., Alsberg E. (2009). Formation of ordered cellular structures in suspension via label-free negative magnetophoresis. Nano Lett..

[108] Akiyama Y., Morishima K. (2012). Label-free ultrarapid spheroid formation in microfluidic chip using magneto-Archimedes effect. Proceedings of the 2012 IEEE 25th International Conference on Micro Electro Mechanical Systems (MEMS).

[109] Datta P., Moolayadukkam S., Chowdhury D., Rayes A., Lee N.S., Sahu R.P., Zhou Q., Puri I.K. (2024). Recent advances and future directions in sonodynamic therapy for cancer treatment. BME Front..

[110] Anil-Inevi M., Delikoyun K., Mese G., Tekin H.C., Ozcivici E. (2021). Magnetic levitation assisted biofabrication, culture, and manipulation of 3D cellular structures using a ring magnet based setup. Biotechnol. Bioeng..

[111] Anil-Inevi M., Delikoyun K., Mese G., Tekin H.C., Ozcivici E. (2021). Axial-circular magnetic levitation assisted biofabrication and manipulation of cellular structures. bioRxiv.

[112] Sarigil O., Anil-Inevi M., Yilmaz E., Ozcelik O., Mese G., Tekin H.C., Ozcivici E. Magnetic levitation-based adipose tissue engineering using horizontal magnet deployment. Proceedings of the 2020 Medical Technologies Congress (TIPTEKNO).

[113] Tasoglu S., Kavaz D., Gurkan U.A., Guven S., Chen P., Zheng R., Demirci U. (2013). Paramagnetic levitational assembly of hydrogels. Adv. Mater..

[114] Tasoglu S., Yu C., Gungordu H., Guven S., Vural T., Demirci U. (2014). Guided and magnetic self-assembly of tunable magnetoceptive gels. Nat. Commun..

[115] Zlotnick H.M., Clark A.T., Gullbrand S.E., Carey J.L., Cheng X.M., Mauck R.L. (2020). Magneto-Driven Gradients of Diamagnetic Objects for Engineering Complex Tissues. Adv. Mater..

[116] Perez J.E., Nagle I., Wilhelm C. (2021). Magnetic molding of tumor spheroids: Emerging model for cancer screening. Biofabrication.

[117] Bilginer-Kartal R., Arslan-Yildiz A. (2025). Magnetic Levitational Assembly of Differentiated SH-SY5Y Cells for Aβ-Induced 3D Alzheimer’s Disease Modeling and Curcumin Screening. Macromol. Biosci..

[118] Kumar C.S., Mohammad F. (2011). Magnetic nanomaterials for hyperthermia-based therapy and controlled drug delivery. Adv. Drug Deliv. Rev..

[119] Paolini A., Guarch C.P., Ramos-López D., de Lapuente J., Lascialfari A., Guari Y., Larionova J., Long J., Nano R. (2016). Rhamnose-coated superparamagnetic iron-oxide nanoparticles: An evaluation of their in vitro cytotoxicity, genotoxicity and carcinogenicity. J. Appl. Toxicol..

[120] Abdel Fattah A.R., Abdalla A.M., Mishriki S., Meleca E., Geng F., Ghosh S., Puri I.K. (2017). Magnetic Printing of a Biosensor: Inexpensive Rapid Sensing to Detect Picomolar Amounts of Antigen with Antibody-Functionalized Carbon Nanotubes. ACS Appl. Mater. Interfaces.

[121] Zhang C., Zhao P., Gu F., Zhang X., Xie J., He Y., Zhou H., Fu J., Turng L.-S. (2020). Axial-Circular Magnetic Levitation: A Three-Dimensional Density Measurement and Manipulation Approach. Anal. Chem..

[122] Iyad N., Ahmad M.S., Alkhatib S.G., Hjouj M. (2023). Gadolinium contrast agents-challenges and opportunities of a multidisciplinary approach: Literature review. Eur. J. Radiol. Open.

[123] Coimbra S., Rocha S., Sousa N.R., Catarino C., Belo L., Bronze-da-Rocha E., Valente M.J., Santos-Silva A. (2024). Toxicity mechanisms of gadolinium and gadolinium-based contrast agents—A review. Int. J. Mol. Sci..

[124] Fattah A.R.A., Mishriki S., Kammann T., Sahu R.P., Geng F., Puri I.K. (2018). Gadopentatic acid affects in vitro proliferation and doxorubicin response in human breast adenocarcinoma cells. BioMetals.

[125] Shukla S., Jadaun A., Arora V., Sinha R.K., Biyani N., Jain V. (2015). In vitro toxicity assessment of chitosan oligosaccharide coated iron oxide nanoparticles. Toxicol. Rep..

[126] Feng Q., Liu Y., Huang J., Chen K., Huang J., Xiao K. (2018). Uptake, distribution, clearance, and toxicity of iron oxide nanoparticles with different sizes and coatings. Sci. Rep..

[127] Li X., Qin S., Liao X., Li Y., Liu A. (2025). Magnetic nanoparticles influence the biological function of mesenchymal stem cells. Sci. Rep..

[128] Freed L.E., Langer R., Martin I., Pellis N.R., Vunjak-Novakovic G. (1997). Tissue engineering of cartilage in space. Proc. Natl. Acad. Sci. USA.

[129] Stamenković V., Keller G., Nesic D., Cogoli A., Grogan S.P. (2010). Neocartilage formation in 1 g, simulated, and microgravity environments: Implications for tissue engineering. Tissue Eng. Part A.

[130] Parfenov V.A., Khesuani Y.D., Petrov S.V., Karalkin P.A., Koudan E.V., Nezhurina E.K., Pereira F.D., Krokhmal A.A., Gryadunova A.A., Bulanova E.A. (2020). Magnetic levitational bioassembly of 3D tissue construct in space. Sci. Adv..

[131] (2021). Global 3D Bioprinting Market Size & Trends Report 2021–2028. https://www.grandviewresearch.com/industry-analysis/3d-bioprinting-market.

[132] Hogan M., Souza G., Birla R. (2016). Assembly of a functional 3D primary cardiac construct using magnetic levitation. AIMS Bioeng..

[133] NIH to Prioritize Human-Based Research Technologies. https://www.nih.gov/news-events/news-releases/nih-prioritize-human-based-research-technologies.

[134] Shahin-Shamsabadi A., Selvaganapathy P.R. (2021). Engineering Murine Adipocytes and Skeletal Muscle Cells in Meat-like Constructs Using Self-Assembled Layer-by-Layer Biofabrication: A Platform for Development of Cultivated Meat. Cells Tissues Organs.

[135] Bilirgen A.C., Toker M., Odabas S., Yetisen A.K., Garipcan B., Tasoglu S. (2021). Plant-Based Scaffolds in Tissue Engineering. ACS Biomater. Sci. Eng..

[136] Temirel M., Hawxhurst C., Tasoglu S. (2021). Shape Fidelity of 3D-Bioprinted Biodegradable Patches. Micromachines.

